# Factors Associated with Anxiety and Depressive Symptoms in 2775 Patients with Arterial Hypertension and Coronary Heart Disease: Results from the COMETA Multicenter Study

**DOI:** 10.5334/gh.1017

**Published:** 2021-10-19

**Authors:** Nana Pogosova, Sergey Boytsov, Dirk De Bacquer, Olga Sokolova, Aza Ausheva, Alexander Kursakov, Hugo Saner

**Affiliations:** 1 National Medical Research Center of Cardiology, Moscow, RU; 2 Department of Public Health and Primary Care, Ghent University, BE; 3 Institute for Social and Preventive Medicine, University of Bern, CH; 4 I.M. Sechenov First Moscow State Medical University, Moscow, RU

**Keywords:** Anxiety symptoms, depressive symptoms, arterial hypertension, coronary heart disease, lifestyle risk factors, stress, psychosocial risk factors

## Abstract

**Aim:**

To identify associations of anxiety symptoms (AS) and depressive symptoms (DS) with other psychosocial and lifestyle risk factors in primary care patients with arterial hypertension (AH) and/or coronary heart disease (CHD).

**Methods:**

COMETA (Clinical-epidemiOlogical prograM of studying psychosocial risk factors in cardiological practice in patiEnts with arterial hyperTension and ischemic heArt disease) is a multicenter cross-sectional study performed in 30 big cities of Russia with two to five out-patient clinics per city randomly selected and two to five general practitioners (GPs) per an out-patient clinic. Each GP included 8–10 consecutive patients with AH and/or CHD. AS and DS were assessed by the Hospital Anxiety and Depression Scale.

**Results:**

325 GPs enrolled 2775 patients (mean age 66.7 years, 72% women) with AH (60.8%), CHD (2.6%), and AH plus CHD (36.6%). Moderate/severe (≥11 HADS) AS were found in 25.5% and DS in 16.3% patients. The strongest associations of AS and DS were revealed for high stress level (OR 5.79; 95% CI [4.18–8.03]), moderate stress level (OR 2.34; 95% CI [1.73–3.16]), low social support (OR 1.87; 95% CI [1.31–2.68]) and female gender (OR 1.78; 95% CI [1.41–2.25]). Low physical activity, unhealthy eating, unemployment and low income were also positively associated with both AS and DS (p < 0.003 for all).

**Conclusion:**

In out-patients with AH and CHD, AS and DS were strongly associated with higher levels of stress, low social support, unemployment, low family income and unhealthy lifestyle such as low physical activity, low fruit and vegetables intake and excessive salt consumption. Our findings indicate that patients with AH and CHD, who have anxiety and depressive symptoms need extra attention and monitoring in regard to stress and lifestyle risk factor control.

## Introduction

Both anxiety and depressive disorders as well as their subclinical symptoms are known as psychosocial risk factors for cardiovascular disease (CVD) in healthy people [1,2], and are also recognized as risk factors for cardiovascular events and mortality in patients with known CVD [3,4]. Moreover, depression may mediate the adverse effects of other psychosocial risk factors such as stressful life events and low social support [5]. Along with a number of plausible biological mechanisms underlying these associations [6,7], depression and anxiety may act as possible barriers for both lifestyle changes and medication adherence in CVD patients [8,9,10,11]. Current European Guidelines on Cardiovascular Prevention in Clinical Practice have a Class II, Level A recommendation for the assessment of psychosocial risk factors including anxiety and depression in individuals with high risk or established CVD [12].

Research of associations between anxiety, depression and other cardiovascular risk factors may help to improve the detection of negative clinical scenarios. The EUROASPIRE III, IV and V surveys [13,14,15] included assessment of anxiety and depressive symptoms and their associations with other cardiovascular risk factors in patients with coronary heart disease (CHD). EUROASPIRE III found associations between depressive symptoms and body mass index (BMI), waist circumference, fasting glucose, and greater frequency of self-reported diabetes. Both anxiety and depressive symptoms were related to a lower incidence of lifestyle modification. EUROASPIRE IV confirmed associations of anxiety and depressive symptoms with a number of traditional risk factors as well as with lower rates of positive lifestyle changes and lower adherence to cardioprotective medications. The obvious strength of the EUROASPIRE surveys is the possibility to obtain a unique Europe-wide snapshot of multiple risk factors and clinical characteristics in CHD patients from a large number of European countries. Nevertheless, these surveys were performed in limited number of participating centers (most academic or tertiary clinics) and limited number of patients in each country. This highlights the need for more widespread and country specific research, moreover anxiety and depressive symptoms may be influenced by cultural differences or by socioeconomic factors [16,17,18,19].

Assessment of psychosocial risk factors including anxiety and depressive symptoms are far from being routine clinical practice and their importance for adherence to a healthy lifestyle seems to be highly underestimated [20,21,22]. This is particularly true for primary care settings. Therefore, the multicenter COMETA study (Clinical-epidemiOlogical prograM of studying psychosocial risk factors in cardiological practice in patiEnts with arterial hyperTension and ischemic heArt disease) was carried out to evaluate the prevalence of psychosocial risk factors in outpatients with common cardiovascular conditions, namely with arterial hypertension (AH) and CHD. Results on prevalence of psychosocial risk factors in the whole cohort of patients with AH and/or CHD, as well as in main study subgroups were published previously [23,24]. The current manuscript presents the results of the COMETA study in regard to associations between anxiety, depressive symptoms and other psychosocial and lifestyle risk factors, comorbidities and medications in a large sample of primary care patients with AH and CHD.

## Methods

COMETA is a multicenter cross-sectional study, carried out from June 2016 to February 2017 in 30 big cities of Russia, located from the very West (Kaliningrad, close to Poland) to the far East (Khabarovsk, close to Japan) of the country, including Astrakhan, Barnaul, Vladimir, Vologda, Voronezh, Ekaterinburg, Kazan, Kaliningrad, Kemerovo, Krasnodar, Krasnoyarsk, Kursk, Lipetsk, Moscow, Moscow Region (Lyubertsy and Lytkarino), Nizhny Novgorod, Novoaltaysk, Novosibirsk, Omsk, Perm, Samara, St. Petersburg, Saratov, Smolensk, Tomsk, Tula, Tyumen, Ufa, Khabarovsk and Cheboksary.

### Patients

In each city, two to five public territorial out-patient clinics were randomly selected. In each out-patient clinic, two to five GPs, who were full-time employees, were also selected randomly (limited only by refusal to participate). Each GP, who agreed to take part in the study, had to enroll within 1–2 working days 8–10 consecutive patients with AH and/or CHD, who came for a routine visit. Patient’s eligibility criteria: age ≥ 55 years, confirmed AH and/or CHD and signed informed consent. Exclusion criteria: current severe acute health conditions or exacerbation of chronic diseases, any diagnosis of mental disorders, drug or alcohol abuse based on the medical records during the previous five years. No specific strategies were used for patients’ attraction, with the exception of informational leaflets from the Russian National Society of Preventive Cardiology, emphasizing the importance of psychosocial risk factors research in CVD patients.

Height and weight were measured in light indoor clothes without shoes. Overweight was defined as a body mass index (BMI) ≥25 – <30 kg/m^2^ and obesity as BMI ≥30 kg/m^2^. Waist circumference was measured with a metal tape horizontally in the mid-axillary line midway between the lowest rim of the rib cage and tip of the hip bone with the patient standing. Abdominal overweight was defined as a waist circumference of ≥80 – <88 cm for women and ≥94 – <102 cm for men and central obesity as a waist circumference of ≥88 cm for women and ≥102 cm for men. Blood pressure (BP) was measured with a sphygmomanometer twice on the right upper arm in a sitting position after at least three minutes’ rest. The mean of two readings was used for analysis. AH was defined as systolic BP ≥140 and/or diastolic BP ≥90 mmHg or being on any antihypertensive medications at the time of the visit. CHD was considered as confirmed in patients with a history of documented myocardial infarction or typical angina coupled with either positive results of non-invasive tests (any kind of stress-test or coronary CT angiography) or angiographic evidence of significant coronary stenosis (≥50%). The study protocol has been approved by the local Ethical Committee.

### Data collection

Patients were asked to complete a questionnaire which included gender, age, data on lifestyle risk factors (smoking, eating habits, alcohol consumption, physical activity level) and psychosocial risk factors (marital and labor status, social support, self-perceived income level, stress level, stressful events during the preceding year). Level of social support was self-reported as low or high (according to personal perception). Level of income was self-defined by participants as low, medium or high. Physical activity level was also self-reported and defined as low, moderate and high (less than 30 min per day; 30–60 min per day; >60 min per day). Self-reported eating habits included consumption of fruits and vegetables (more or less than 500 g per day), fish (more or less once a week), adding salt to cooked food, restricting saturated fat, and drinking alcohol (yeas or no, and the number of standard drinks per day and per month). The stress level was assessed by means of the Visual Analog Scale (VAS) according to question ‘Please grade the level of constant stress or tension in your life over the preceding year using the 10-point scale below,’ and participant had to mark one number on the scale (0 to 10 VAS). VAS score <4 was considered as low, 5–7 – moderate, and ≥8 – high stress level. The same way of stress assessment was earlier used in the COORDINATA study [25], which revealed strong associations between the VAS score and stressful life events during the preceding year in a similar group of patients. Anxiety and depressive symptoms were assessed by the Hospital Anxiety and Depression Scale (HADS) [26]. A score of 8–10 points on HADS-A and HADS-D subscales was considered as subclinical anxiety and depressive symptoms, and a score of ≥11 points – moderate/severe anxiety and depressive symptoms. Patients were given verbal instructions on how to fill out all questionnaires. They completed the questionnaires on their own in recreational areas of out-patient clinics and returned them to the medical staff.

For each patient, the enrolling GP completed a questionnaire with data on patient’s comorbidities, prior revascularization procedures (both coronary and carotid), average number of angina episodes if present, prescribed medications for AH and CHD, psychotropic drug use, hospitalizations during the preceding year, physical exam data (height, weight, waist circumference, blood pressure), and latest available results of serum cholesterol und glucose during the preceding year based on the medical records data.

## Statistical Analysis

Distributions of patient characteristics were summarized using means and standard deviations for continuous variables and numbers and proportions for categorical variables. Logistic regression analysis was applied to investigate factors associated with symptoms of anxiety and depression independently of age, gender and educational level. Associations were expressed as adjusted odds ratios and their 95% confidence intervals. P-values were based on Wald chi-square statistics. A stepwise approach was applied to obtain an independent set of risk factors for both outcomes. Models assumptions were checked by graphical analyses of residuals. A double-sided type I error level of α = 0·05 was used to indicate statistical significance. Data analyses were undertaken using SAS statistical software (release 9.4) in the Department of Public Health and Primary Care, Ghent University, Belgium.

## Results

Three hundred and twenty-five GPs from 30 big cities, who agreed to participate in the study, enrolled 2775 patients with AH and or CHD, aged 55 to 96 years (mean (SD) 66.7 (7.9) years, 72 % women). The patients’ response rate was 68.2%. Recruiting diagnoses were AH in 1687 patients (60.8%), AH and CHD in 1015 patients (36.6 %) and CHD without AH in 73 patients (2.6%). The prevalence of socioeconomic risk factors was high in the study cohort including 24.5% with low level of education, 25.2% living alone, and 44.2% of patients who perceived their income as low or very low.

According to the HADS questionnaire, subclinical anxiety symptoms (HADS-A 6–10) were found in 21.7% and subclinical depressive symptoms (HADS-D 6–10) – in 26.2%, whereas moderate to severe symptoms of anxiety (HADS-A ≥11) existed in 25.5% and moderate to severe symptoms of depression (HADS-D >11) – in 16.3% of patients. More than two–thirds (66.5%) of patients were free from both anxiety and depressive moderate to severe symptoms, and 8.3% participants had coexisting moderate to severe anxiety and depressive symptoms.

### Relation between anxiety/depressive symptoms and sociodemographic characteristics

Table 1 presents the distribution of anxiety and depressive symptoms in relation to sociodemographic characteristics of patients. After adjustment for gender, age and educational level, moderate/severe anxiety symptoms were more common in women, less educated people, participants with low and very low self-perceived family income, low social support, higher stress level (45.6% vs 10.7%) and in patients with a registered disability. After the same adjustment, moderate/severe depressive symptoms were more prevalent in older age groups, less educated and unemployed people, patients with low and very low family income (21.9% vs 11.9%), low social support (35.1% v 14.9%), high stress level (25.4% vs 10%) and registered disability.

**Table 1:** Anxiety and depressive symptoms in relation to sociodemographic characteristics of patients.

Sociodemographic characteristics	Level	Symptoms of anxiety		Symptoms of depression	

		HADS-A score ≥ 8 to 10, % (n/N)	HADS-A score ≥ 11, % (n/N)	HADS-D score ≥ 8 to 10, % (n/N)	HADS-D score ≥ 11, % (n/N)

Gender	Women	23.4% (464/1984)	28.7% (570/1984)	26.5% (526/1984)	17.2% (342/1984)
	Men	17.3% (134/774)	17.3% (134/774)**	25.3% (196/774)	13.8% (107/774)
Age	<60 years	19.3% (105/545)	23.1% (126/545)	23.3% (127/545)	12.3% (67/545)
	60–69 years	23.0% (312/1359)	24.2% (329/1359)	25.8% (350/1359)	13.6% (185/1359)
	70–79 years	22.9% (149/650)	27.7% (180/650)	28.5% (185/650)	20.9% (136/650)**
	≥80 years	15.7% (32/204)	33.8% (69/204)	29.4% (60/204)	29.9% (61/204)**
Level of education	Higher education	19.6% (175/893)	22.8% (204/893)	22.5% (201/893)	14.1% (126/893)
	In between	22.8% (275/1205)	25.1% (302/1205)	28.1% (339/1205)	14.7% (177/1205)
	Secondary or less	22.4% (146/653)	30.2% (197/653)*	27.4% (179/653)	22.2% (145/653)**
Labor status	Working	21.1% (160/757)	20.7% (157/757)	23.5% (178/757)	9.4% (71/757)
	Not working^1^	21.8% (433/1987)	27.4% (544/1987)	27.1% (539/1987)	18.9% (376/1987)**
Family income	Higher	20.1% (308/1531)	22.2% (340/1531)	23.4% (358/1531)	11.9% (182/1531)
	Low or very low	23.4% (284/1213)	29.8% (362/1213)**	29.6% (359/1213)	21.9% (265/1213)**
Living situation	With family members	21.4% (437/2039)	24.7% (503/2039)	26.1% (532/2039)	15.3% (311/2039)
	Living alone	22.6% (154/683)	28.1% (192/683)	25.8% (176/683)	19.5% (133/683)
Social support	High	21.7% (558/2570)	24.0% (616/2570)	25.8% (664/2570)	14.9% (382/2570)
	Low	20.7% (36/174)	47.7% (83/174)**	31.0% (54/174)	35.1% (61/174)**
Stress level	Low (VAS score < 4)	14.0% (84/600)	10.7% (64/600)	20.0% (120/600)	10.3% (62/600)
	Moderate (VAS score 4–7)	24.5% (382/1557)	23.8% (370/1557)	27.8% (432/1557)	15.2% (237/1557)
	High (VAS score ≥ 8)	22.0% (130/590)	45.6% (269/590)**	27.8% (164/590)	25.4% (150/590)**
Registered disability	No	20.9% (363/1734)	23.8% (413/1734)	24.5% (425/1734)	13.3% (230/1734)
	Yes	22.8% (227/996)	28.5% (284/996)*	28.9% (288/996)	21.4% (213/996)**

Statistical significance after adjustment for gender, age and educational level (for HADS scores ≥ 11 only): * P < 0.01; ** P < 0.001. ^1^ – Among not working 97% retired and 3% unemployed.

### Relation between anxiety/depressive symptoms and lifestyle risk factors

Symptoms of anxiety and depression were unequally distributed among patients with different lifestyle habits (Table 2). After adjustment for gender, age and education, moderate/severe anxiety symptoms were significantly more prevalent in those who denied drinking alcohol at least occasionally (28.7% vs 19.8%), patients with low level of physical activity, in those not restricting salt (30.1% vs 23.5%) and those, who reported low fruit, vegetables and fish intake. High scores of depressive symptoms were twice more common in patients abstaining from alcohol (19.5% vs 10.5%), walking less than 30 minutes per day (26.9% vs 12–15% in other subgroups) and eating less fruit, vegetables and fish. Smoking status, BMI, waist circumference and saturated fat consumption showed no relation with anxiety and depression symptoms.

**Table 2:** Anxiety and depressive symptoms in relation lifestyle risk factors.

Lifestyle characteristic	Level	Symptoms of anxiety		Symptoms of depression	

		HADS-A score ≥ 8 to 10, % (n/N)	HADS-A score ≥ 11, % (n/N)	HADS-D score ≥ 8 to 10, % (n/N)	HADS-D score ≥ 11, % (n/N)

Smoking status	Never smoked	22.8% (480/2108)	27.2% (573/2108)	26.2% (552/2108)	17.1% (360/2108)
	Former smoker	19.4% (78/403)	17.6% (71/403)	25.3% (102/403)	13.2% (53/403)
	Current smoker	15.6% (36/231)	23.8% (55/231)	26.8% (62/231)	14.7% (34/231)
Alcohol consuption	No	22.4% (390/1741)	28.7% (499/1741)	27.7% (482/1741)	19.5% (340/1741)
	Yes	20.2% (200/990)	19.8% (196/990)**	23.2% (230/990)	10.5% (104/990)**
Physical activity	>60 min/day	22.0% (245/1114)	23.5% (262/1114)	22.4% (250/1114)	12.1% (135/1114)
	30 to 60 min/day	21.8% (235/1078)	22.3% (240/1078)	26.3% (283/1078)	15.1% (163/1078)
	<30 min/day	21.0% (117/557)	35.6% (198/557)**	33.8% (188/557)	26.9% (150/557)**
Body mass index	<25 kg/m^2^	21.1% (109/516)	23.8% (123/516)	26.4% (136/516)	17.1% (88/516)
	25–29 kg/m^2^	22.0% (246/1118)	24.8% (277/1118)	24.3% (272/1118)	15.7% (175/1118)
	30–35 kg/m^2^	22.5% (161/717)	24.4% (175/717)	28.0% (201/717)	14.5% (104/717)
	≥35 kg/m^2^	19.5% (72/370)	31.9% (118/370)	26.5% (98/370)	20.0% (74/370)
Waist circumference#	Normal	21.4% (130/608)	23.0% (140/608)	24.7% (150/608)	16.5% (100/608)
	Abdominal overweight	19.1% (103/538)	25.3% (136/538)	23.6% (127/538)	14.5% (78/538)
	Central obesity	23.0% (337/1467)	27.0% (396/1467)	27.7% (407/1467)	17.3% (254/1467)
Salt added to cooked food	No	21.9% (412/1883)	23.5% (443/1883)	25.1% (472/1883)	15.9% (300/1883)
	Yes	21.2% (181/855)	30.1% (257/855)**	28.3% (242/855)	17.4% (149/855)
Fruit & vegetable consumption	High (≥500 g)	21.2% (359/1690)	21.2% (359/1690)	24.1% (407/1690)	12.4% (209/1690)
	Low (<500 g)	22.5% (237/1052)	32.2% (339/1052)**	29.2% (307/1052)	22.8% (240/1052)**
Fish consumption	At least once a week	20.9% (233/1116)	21.5% (240/1116)	22.1% (247/1116)	13.9% (155/1116)
	Less than once a week	22.4% (364/1626)	28.1% (457/1626)**	28.7% (467/1626)	18.1% (294/1626)*
Saturated fat consumption	Not restricted	21.2% (170/803)	26.9% (216/803)	27.2% (218/803)	16.4% (132/803)
	Restricted	21.8% (418/1915)	25.0% (478/1915)	25.9% (495/1915)	16.2% (310/1915)

Statistical significance after adjustment for gender, age and educational level (for HADS scores ≥ 11 only): * P < 0.01; ** P < 0.001. # Abdominal overweight was defined as waist circumference of ≥80 cm in females and ≥94 cm in males, and females with waist circumference of ≥88 cm or males with waist circumference of ≥102 cm were diagnosed with central obesity.

### Relation between anxiety/depressive symptoms and clinical findings

We also evaluated the rates of anxiety and depressive symptoms according to patients’ diagnosis (AH or CHD) and other clinical characteristics collected from personal medical records (Table 3). Moderate/severe depressive symptoms were significantly more common in patients with CHD as compared to AH (19.9% vs 14.0%), whereas high anxiety scores were similarly prevalent in approximately one in four patients from both diagnostic categories. A history of MI or coronary revascularization did not affect the rates either for anxiety or for depressive symptoms. Clinically significant depressive symptoms were more prevalent in patients with exertional angina, conduction anomalies and diabetes. Both anxiety and depressive symptoms were more prevalent in patients with any premature beats, but not with atrial fibrillation. Neither anxiety, nor depressive symptoms showed any specific patterns in heart failure patients. Prior diagnosis of cerebrovascular diseases and stroke did not seem to be connected with depressive symptoms, although participants with cerebrovascular diseases (except stroke) had higher rates of anxiety symptoms.

**Table 3:** Anxiety and depressive symptoms in relation to manifestations of CVD.

Comorbidity	Level	Symptoms of anxiety		Symptoms of depression	

		HADS-A score ≥ 8 to 10, % (n/N)	HADS-A score ≥ 11, % (n/N)	HADS-D score ≥ 8 to 10, % (n/N)	HADS-D score ≥ 11, % (n/N)

Inclusion criteria	Hypertension	22.7% (380/1678)	24.7% (415/1678)	26.2% (440/1678)	14.0% (234/1678)
	CHD	20.2% (218/1080)	26.8% (289/1080)	26.1% (282/1080)	19.9% (215/1080)**
History of MI	No	22.3% (534/2392)	25.7% (615/2392)	25.9% (619/2392)	16.6% (397/2392)
	Yes	17.5% (64/366)	24.3% (89/366)	28.1% (103/366)	14.2% (52/366)
History of revascularization	No	22.0% (544/2473)	25.9% (640/2473)	25.7% (636/2473)	16.9% (417/2473)
	Yes	19.0% (54/285)	22.5% (64/285)	30.2% (86/285)	11.2% (32/285)
Angina at rest	No	21.6% (581/2693)	25.4% (684/2693)	26.0% (701/2693)	16.1% (434/2693)
	Yes	22.8% (13/57)	33.3% (19/57)	31.6% (18/57)	22.8% (13/57)
Exertional angina	No	21.8% (468/2151)	24.4% (525/2151)	26.7% (574/2151)	14.7% (317/2151)
	Yes	21.4% (130/607)	29.5% (179/607)	24.4% (148/607)	21.8% (132/607)*
Diabetes (Type 1 or 2)	No	21.5% (456/2125)	25.3% (537/2125)	25.5% (542/2125)	15.0% (319/2125)
	Yes	22.4% (139/622)	26.1% (162/622)	28.5% (177/622)	20.4% (127/622)*
Extrasystoles	No	22.1% (475/2146)	23.6% (507/2146)	26.1% (561/2146)	14.8% (318/2146)
	Yes	20.0% (120/601)	32.0% (192/601)**	26.3% (158/601)	21.3% (128/601)**
Atrial fibrillation	No	21.4% (533/2497)	25.4% (633/2497)	26.3% (656/2497)	15.6% (389/2497)
	Yes	24.8% (62/250)	26.4% (66/250)	25.2% (63/250)	22.8% (57/250)
Conduction disturbances	No	21.5% (537/2497)	24.9% (622/2497)	26.4% (658/2497)	15.3% (383/2497)
	Yes	23.2% (58/250)	30.8% (77/250)	24.4% (61/250)	25.2% (63/250)*
Heart failure	No	22.1% (385/1743)	23.9% (417/1743)	23.8% (415/1743)	14.1% (245/1743)
	Yes	20.9% (210/1004)	28.1% (282/1004)	30.3% (304/1004)	20.0% (201/1004)
Cerebrovascular disease	No	20.4% (282/1380)	21.7% (299/1380)	23.0% (318/1380)	13.3% (183/1380)
	Yes	22.9% (313/1367)	29.3% (400/1367)*	29.3% (401/1367)	19.2% (263/1367)
Prior stroke	No	21.5% (552/2572)	25.3% (651/2572)	26.2% (673/2572)	15.8% (406/2572)
	Yes	24.6% (43/175)	27.4% (48/175)	26.3% (46/175)	22.9% (40/175)

Statistical significance after adjustment for gender, age and educational level (for HADS scores ≥ 11 only): * P < 0.01; ** P < 0.001.

Comorbidities such as back pain, chronic respiratory diseases and chronic kidney disease had no specific patterns in regard to moderate/severe anxiety or depressive symptoms with exception of moderate/severe anxiety, which was more prevalent in patients with peptic ulcers (35.9% vs 24.8%, p < 0.001) and back pain (27.5% vs 21.7%, p < 0.01).

### Relation of anxiety/depressive symptoms with medications

Overall, 60.2% of patients with AH and CHD received aspirin, 61.5% statins, 81.9% ACE inhibitors, 58.9% beta-blockers, 26.3% calcium channel blockers, 50.3% diuretics, 7.3% anticoagulants and 4.3% glucose lowering drugs. The anxiety and depressive symptoms prevalence was not related to the majority of drug classes commonly prescribed for AH and CHD with the only exception for statins. HADS-A scores of ≥11 was seen in 27.4% of patients receiving statins compared with 22.7% who did not (p < 0.01). High HADS-D scores occurred more often in patients on nitrates (24.0% vs 15.3%). As for the psychotropic drugs, a larger proportion of patients on anxiolytics had high scores of both anxiety and depressive symptoms (48.5% vs 24.1% and 30.6% vs 15.6%, both p < 0.001). And a larger proportion of patients on antidepressants had moderate to severe depressive symptoms (41.0% vs 16.6%).

### Prevalence of lifestyle and socioeconomic factors associated with anxiety/depressive symptoms

The results of multivariate stepwise logistic regression analyses of factors associated with anxiety symptoms are shown in Table 4 and with depressive symptoms in Table 5. The most prominent factor associated with moderate/severe anxiety symptoms was self-reported stress: high level of stress (≥8 VAS points) increased the odds of anxiety by nearly 6 times, moderate level of stress (4–7 VAS points) – by 2.34 times (p < 0.0001). Stress was followed by low social support (OR 1.87; 95% CI [1.31 to 2.68], p = 0.0007), female gender (OR 1.78; 95% CI [1.41 to 2.25], p < 0.0001), low level of physical activity (OR 1.55; 95% CI [1.20 to 2.01], p = 0.0007), low fruit and vegetable consumption (OR 1.50; 95% CI [1.23 to 1.84], p < 0.0001), being unemployed (OR 1.43; 95% CI [1.14 to 1.79], p = 0.0021) and adding salt to cooked food (OR 1.41; 95% CI [1.17 to 1.76], p = 0.0005). Moderate alcohol intake was inversely associated with anxiety symptoms (OR 0.73; 95% CI [0.59 to 0.90], p = 0.0028).

**Table 4:** Factors associated with anxiety symptoms: results of a multivariate stepwise logistic regression analysis.

	Outcome: HADS - Anxiety score ≥ 11	

	Adjusted Odds Ratio (95% CI)	P-value

High stress level (VAS score ≥ 8)	5.79 (4.18 to 8.03)	P < 0.0001
Moderate stress level (VAS score 5–7)	2.34 (1.73 to 3.16)	P < 0.0001
Low social support	1.87 (1.31 to 2.68)	P = 0.0007
Female gender	1.78 (1.41 to 2.25)	P < 0.0001
Walking < 30 min/day	1.55 (1.20 to 2.01)	P = 0.0007
Low fruit and vegetable consumption	1.50 (1.23 to 1.84)	P < 0.0001
Unemployed	1.43 (1.14 to 1.79)	P = 0.0021
Adding salt to cooked food	1.41 (1.17 to 1.76)	P = 0.0005
Use of alcohol	0.73 (0.59 to 0.90)	P = 0.0028

*Note*: In the stepwise logistic regression model described above, the following factors were included in the models: gender, age, educational level, family income, social support, stress level, registered disability, drinking alcohol, walking, salt added to cooked food, fruit & vegetables consumption, fish consumption, cerebrovascular disease, extra systoles, back pain, peptic ulcer and statins.

**Table 5:** Factors associated with depressive symptoms: results of a multivariate stepwise logistic regression analysis.

	Outcome: HADS - Depression score ≥ 11	

	Adjusted Odds Ratio (95% CI)	P-value

High stress level (VAS score ≥ 8)	2.28 (1.60 to 3.23)	P < 0.0001
Walking < 30 min/day	2.03 (1.51 to 2.72)	P < 0.0001
Low social support	1.87 (1.28 to 2.74)	P = 0.0012
Use of alcohol	0.57 (0.44 to 0.74)	P < 0.0001
Unemployed	1.73 (1.29 to 2.34)	P = 0.0003
Low fruit and vegetable consumption	1.68 (1.33 to 2.11)	P < 0.0001
Low family income	1.66 (1.32 to 2.09)	P < 0.0001
Registered disability	1.53 (1.21 to 1.93)	P = 0.0003

*Note*: In the stepwise logistic regression model described above, the following factors were included in the models: gender, age, educational level, employment, family income, social support, stress level, registered disability, drinking alcohol, walking, fruit & vegetables consumption, fish consumption, inclusion criteria, exertional angina, diabetes (Type I or II), extra systoles, conduction disturbances, and nitrates.

The leading factor associated with moderate/severe depressive symptoms was also high level of stress (OR 2.28; 95% CI [1.60 to 3.23], p < 0.0001). Low level of physical activity increased the odds of moderate/severe depressive symptoms two times (OR 2.03; 95% CI [1.51 to 2.72], p < 0.0001) and was followed by low social support (OR 1.87; 95% CI [1.28 to 2.74], p = 0.0012), being unemployed (OR 1.73; 95% CI [1.29 to 2.34], p = 0.0003), low fruit and vegetables consumption (OR 1.68; 95% CI [1.33 to 2.11], p < 0.0001), low family income (OR 1.66; 95% CI [1.32 to 2.09], p < 0.0001) and registered disability (OR 1.53; 95% CI [1.21 to 1.93], p = 0.0003). As with anxiety, moderate alcohol intake was inversely associated with depressive symptoms, having reduced the risk nearly by a half (OR 0.57; 95% CI [0.44 to 0.74], p < 0.0001).

## Discussion

Our results confirm that moderate/severe anxiety and depressive symptoms are strongly associated with a broad range of other psychosocial and lifestyle risk factors in primary care patients with AH and CHD. The leading factor related to moderate/severe anxiety and depressive symptoms was a high level of stress. It is well known that negative effects of stress, anxiety and depression on the cardiovascular system are mediated through immune and neuroendocrine pathways [6,7]. Our findings are of particular importance because they indicate that stress may exert its negative effects on the cardiovascular system to a great extend through stress related poor health behavior.

Compared to the COORDINATA study [25], with a similar design, performed 10 years ago, the COMETA study showed a substantially lower prevalence of anxiety and depressive symptoms in primary care patients with AH and CHD. The prevalence of anxiety symptoms decreased from 35.5% to 25.5% and depressive symptoms from 35.3% to 16.3%. One explanation could be a positive socioeconomic trend in the country over the past decade [27]. However, the rates are considerably higher than in the Russian cohort of patients with CHD in the EUROASPIRE IV survey [14]. Plausible explanations for this difference could be substantially wider geographical area of current study and the abovementioned limitations of the EUROASPIRE.

According to the multivariate stepwise logistic regression analysis, the most prominent factor associated with both anxiety and depressive symptoms was high level of stress. There are numerous reports with similar findings regarding role of perceived stress for anxiety and depression in different groups, for instance in healthy medical students or in women with polycystic ovary syndrome [28,29]. Other relevant factors both for anxiety and depressive symptoms comprised low social support, unemployment and unhealthy behavior, like walking less than 30 minutes per day and low fruit and vegetables consumption. As for low social support, several reports are available linking it to both anxiety and depressive symptoms in many clinical settings including general population [30], patients receiving psychiatric treatment [31], patients with chronic medical conditions [32,33,34]. Swedish researchers have shown that social support and higher household income were strongly related with improved emotional status (namely, less anxiety and depressive symptoms) at long-term follow-up after cardiac rehabilitation [34]. In our study, low family income was associated only with depressive symptoms, but not with anxiety. With respect to unemployment, an extensive review of its negative health sequelae was shown by B. Herbig et al. [35]: the long-term effects of unemployment included increased risk of both depressive and anxiety symptoms along with other mental health problems.

The association of anxiety, depressive symptoms and lifestyle risk factors in CHD patients was extensively studied in the EUROASPIRE IV study [14], where the level of physical activity was inversely related with both anxiety and depressive symptoms, similarly to our study.

At the same time unlike EUROASPIRE IV [14], being female in our study independently increased the odds of anxiety but not depressive symptoms. Given the abundance of data showing a greater prevalence of both anxiety and depression in women it seems that this discrepancy may be a result of screening [36].

Another interesting finding was an independent inverse association of alcohol intake with both anxiety and depressive symptoms. This is somewhat contrary to the available literature, which indicates that alcohol consumption aggravates the existing mental disorders, and moderate consumption is neutral at best [37,38,39]. In this regard, it has to be pointed out that the participants of our study consumed alcohol quite rarely indeed. The reported intake rates were 1.4 ± 1.0 and 2.5 ± 2.4 times per month in women and men, respectively [23]. This corresponds to the consumption of alcohol on holidays and other special occasions only, which is typical for our elderly population. At the same time, the volume of consumed alcohol was close to what is usually considered moderate (1.7 ± 1.4 standard drinks per day in women and 2.3 ± 1.6 standard drinks per day in men).

COMETA study has a number of limitations. The assessment of numerous risk factors, e.g. smoking, dietary habits, stress level was self-reported, which raises the possibility of bias. Another obvious limitation is the cross-sectional design of the study which does not allow conclusions in regard to causality between analyzed factors. Anxiety and depression may be causes but effects as well. Moreover, anxiety and depressive symptoms were assessed by means of HADS screening tool only and did not include assessments by mental health professionals. Finally, it should be noted that the majority of participants were women, and the obtained results may not reflect the situation in men. Nevertheless, this situation is typical for primary care settings, especially in our country, since women are more likely to seek for medical care and women predominate in older age groups due their longer life expectancy [40,41,42].

## Conclusion

In primary care out-patients with AH and CHD, anxiety and depressive symptoms were strongly associated with higher levels of stress, low social support, unemployment, low self-reported family income and unhealthy lifestyle such as low physical activity, low fruit and vegetables intake and excessive salt consumption. Our findings indicate that patients with AH and CHD, who have anxiety and depressive symptoms need extra attention and monitoring in regard to lifestyle risk factors control.
